# Plasma monocyte chemoattractant protein-1 and the risk of kidney and cardiovascular outcomes in people with chronic kidney disease: results from the BRIGHTEN study

**DOI:** 10.1186/s12882-025-04633-y

**Published:** 2025-12-29

**Authors:** Tadashi Toyama, Tatsuo Kagimura, Kenichiro Tanabe, Yasunori Iwata, Takashi Wada, Ichiei Narita

**Affiliations:** 1 Department of Nephrology, Faculty of Medical Sciences, University of Fukui, Yoshida-gun, Japan; 2 Department of Nephrology and Rheumatology, Kanazawa University, Kanazawa, Japan; 3 Translational Research Center for Medical Innovation, Kobe, Japan; 4 Department of Frontier Medicine, St. Marianna University School of Medicine, Kawasaki, Japan; 5 Division of Clinical Nephrology and Rheumatology, Kidney Research Center, Niigata University Graduate School of Medical and Dental Sciences, Niigata, Japan

**Keywords:** Cardiovascular events, Chronic kidney disease, Kidney function decline, Monocyte chemoattractant protein-1, Risk prediction

## Abstract

**Background:**

Monocyte chemoattractant protein-1 (MCP-1) has been implicated in the pathogenesis of chronic kidney disease (CKD); however, its clinical significance in CKD remains unclear. This study investigated the association between plasma MCP-1 levels and kidney and cardiovascular outcomes in patients with CKD.

**Methods:**

This multicenter, prospective, observational analysis used data from the BRIGHTEN study. Participants with anemia and CKD (eGFR < 60 mL/min/1.73 m^2^) were classified into four groups based on quartiles of their baseline plasma MCP-1 levels. The primary outcomes included kidney outcomes (a composite of initiation of maintenance dialysis, kidney transplantation, 50% decline in eGFR, or eGFR ≤ 6 mL/min/1.73 m^2^), the annual rate of eGFR decline, cardiovascular outcomes, and all-cause mortality. Associations were assessed using Cox proportional hazard and linear mixed-effects models.

**Results:**

This study included 1,447 participants with advanced CKD (median eGFR 17.9 mL/min/1.73 m²). The group with the lowest MCP-1 level (< 324.5 pg/mL) had the highest baseline eGFR. In the univariable analyses, higher MCP-1 levels were associated with an increased risk of kidney and cardiovascular outcomes (log-rank *p* < 0.01 and *p* = 0.03, respectively), although these associations were not significant after multivariable adjustment. Analysis of the annual eGFR decline using a multivariable linear mixed-effects model revealed that higher MCP-1 levels were associated with an additional eGFR decline of approximately 1.0 mL/min/1.73 m^2^ per year compared to the lowest MCP-1 group. MCP-1 levels were not associated with mortality.

**Conclusions:**

In patients with CKD, higher plasma MCP-1 levels are associated with a more rapid decline in kidney function but not with the composite outcome of kidney failure. MCP-1 was not associated with cardiovascular outcomes or mortality in this population cohort.

**Supplementary Information:**

The online version contains supplementary material available at 10.1186/s12882-025-04633-y.

## Introduction

Chronic kidney disease (CKD) is a growing global health concern associated with an increased risk of end-stage kidney disease, cardiovascular events, and mortality [1]. CKD progression is complex and involves multiple pathophysiological processes, including inflammation [2], fibrosis [3], and oxidative stress [4]. Identifying novel biomarkers that predict CKD progression and aid in risk stratification is crucial for improving patient outcomes and optimizing disease management [5].

Monocyte chemoattractant protein-1 (MCP-1), also known as C-C motif chemokine ligand 2 (CCL2), is a key chemokine that regulates monocyte migration and infiltration [6]. MCP-1 has been implicated in the pathogenesis of various inflammatory and fibrotic diseases, including CKD and cardiovascular diseases [7, 8]. In kidney disease, MCP-1 contributes to tubulointerstitial injury by recruiting and activating monocytes/macrophages, which are associated with disease progression [9, 10]. Additionally, population-based studies have identified MCP-1 as a predictor of cardiovascular mortality [11], with an association between elevated MCP-1 levels and increased mortality risk [12, 13]. However, comprehensive evidence regarding the role of circulating MCP-1 levels in CKD progression, cardiovascular events, and overall survival is limited.

This study aimed to evaluate the association between plasma MCP-1 levels and kidney and cardiovascular outcomes, as well as all-cause mortality, in patients with CKD. We hypothesized that higher MCP-1 levels are associated with CKD progression, adverse cardiovascular events, and increased mortality. Understanding these associations may help identify high-risk patients with CKD who could benefit from close monitoring and targeted therapeutic interventions.

## Materials and methods

### Study design and participants

This multicenter, prospective, observational study analyzed data from the BRIGHTEN study, the design and rationale of which have been previously described [14, 15]. Briefly, the BRIGHTEN study was conducted at 168 centers across Japan, enrolling 1,695 participants with anemia and CKD (estimated glomerular filtration rate [eGFR] < 60 mL/min/1.73 m^2^) who were initiating darbepoetin alfa treatment for the first time. For this analysis, we included participants with available baseline plasma MCP-1 levels.

Participants were ESA-naïve patients who initiated darbepoetin alfa at 30 µg every 2 weeks within 8 weeks of registration, with dose adjustments (30–180 µg) at physician discretion to maintain Hb ≥ 11 g/dL.

### Exposure and outcomes

Baseline plasma MCP-1 levels were measured before darbepoetin alfa initiation using a quantitative sandwich enzyme-linked immunosorbent assay (Quantikine^®^ Human CCL2/MCP-1 Immunoassay, R&D Systems, Minneapolis, MN, USA). Each assay included a standard curve generated from kit-supplied recombinant human MCP-1 standards. The manufacturer-reported intra-assay coefficient of variation ranged from 4.2% to 7.8%. Plasma samples were collected at a single time point at baseline. Participants were stratified into four groups based on quartiles of baseline MCP-1 levels (Q1: <25th percentile; Q2: 25th to < 50th percentile; Q3: 50th to < 75th percentile; Q4: ≥75th percentile). The primary outcomes included: (1) kidney outcomes, defined as a composite of maintenance dialysis initiation, kidney transplantation, a 50% eGFR decline from baseline, or eGFR ≤ 6 mL/min/1.73 m^2^; (2) annual eGFR decline rate; (3) cardiovascular outcomes; and (4) all-cause mortality.

Baseline plasma MCP-1 levels were measured before darbepoetin alfa initiation. Serum creatinine was measured at baseline and at weeks 2, 4, 6, 8, 10, 12, 16, 24, 36, 48, 60, 72, 84, and 96 after darbepoetin alfa initiation. Clinical outcomes, including dialysis initiation, kidney transplantation, cardiovascular events, and deaths, were recorded by site investigators through medical record review at each participating center during regular follow-up visits.

Cardiovascular outcomes were defined as a composite of fatal events (including death due to myocardial infarction, congestive heart failure, and cerebrovascular diseases) and non-fatal events (including hospitalization due to myocardial infarction, congestive heart failure, and ischemia of major organs other than the heart and brain, such as ischemic colitis), as detailed in the study protocol [14].

### Statistical analysis

Baseline characteristics were compared across the MCP-1 groups using descriptive statistics. Continuous variables are presented as mean and standard deviation or median with interquartile range, and categorical variables are summarized as frequencies and percentages.

Kaplan–Meier curves and log-rank tests were used to assess the association between MCP-1 levels stratified into quartiles and the risk of kidney, cardiovascular, and mortality outcomes. Cox proportional hazard models were used for multivariable analyses to estimate hazard ratios (HRs) and 95% confidence intervals (CIs) for these outcomes. Annual changes in eGFR were analyzed using linear mixed-effects models that incorporated individual participants as random effects. These multivariable models were adjusted for age, sex, hemoglobin level, systolic blood pressure, body mass index, history of cardiovascular disease, and the urinary protein-creatinine ratio.

Receiver operating characteristic (ROC) curve analysis was used to evaluate the predictive ability of MCP-1 for kidney outcomes, with the MCP-1 cut-off determined using Youden’s index. Net reclassification improvement statistics based on the category-free method further assessed the predictive ability of MCP-1 for adverse kidney outcomes. Integrated Discrimination Improvement statistics were used to assess the incremental predictive value of adding MCP-1 to the baseline models [16].

Subgroup analyses were performed to examine the consistency of the association between MCP-1 levels and kidney outcomes across participant characteristics, including age, sex, smoking status, diabetes, CKD type, history of cardiovascular disease, eGFR, proteinuria, and use of renin-angiotensin system inhibitors.

All statistical analyses were conducted using SAS software (version 9.4; SAS Institute, Cary, NC, USA) and R software (version 4.2.3; R Foundation for Statistical Computing, Vienna, Austria). Statistical significance was defined as a two-sided p-value of < 0.05.

## Results

### Baseline characteristics

Our study included 1,447 participants, whose baseline characteristics are shown in Table 1. The median eGFR was 17.9 mL/min/1.73 m². The distribution of MCP-1 values is shown in Additional File Fig. 1, with a median value of 385.9 pg/mL. Patients with higher baseline eGFR had lower MCP-1 levels (Spearman’s *r* = − 0.14, *p* < 0.001; Additional File Fig. 2). The median follow-up period was 2.35 years (interquartile range: 1.90–2.99 years). During follow-up, 577, 143, and 118 participants experienced kidney, cardiovascular, and all-cause mortalities, respectively. The proportion of men was 58.7%, and the mean age was 69.8 years old. Major risk factors, including diabetes, cardiovascular disease, and proteinuria, were similarly distributed across the MCP-1 groups. However, there was a trend toward a higher smoking prevalence and elevated high-sensitivity C-reactive protein levels in participants with higher MCP-1 levels.

**Table 1:** Baseline participant characteristics according to baseline MCP-1 levels

		Baseline MCP-1 levels (pg/mL)			
	Total (*N* = 1447)	Q1 (< 324.5) (*N* = 361)	Q2 (324.5–<385.9) (*N* = 362)	Q3 (385.9–<461.9) (*N* = 362)	Q4 (≥ 461.9) (*N* = 362)
Baseline MCP-1, pg/mL	385.9 (324.5, 461.9)	289.2 (264.5, 307.4)	353.8 (340.6, 369.9)	419.1 (403.4, 435.9)	530.1 (491.0, 589.5)
Age, years	69.8 (12.0)	68.8 (13.2)	69.6 (12.2)	70.2 (11.3)	70.7 (11.0)
Men, n (%)	849 (58.7%)	194 (53.7%)	214 (59.1%)	219 (60.5%)	222 (61.3%)
Etiology of CKD, n (%)					
Diabetic nephropathy	394 (27.2%)	97 (26.9%)	101 (27.9%)	104 (28.3%)	92 (25.4%)
Chronic glomerulonephritis	321 (22.2%)	80 (22.2%)	91 (25.1%)	97 (21.3%)	73 (20.2%)
Nephrosclerosis	333 (23.0%)	80 (22.2%)	74 (20.4%)	91 (25.1%)	88 (24.3%)
Polycystic kidney disease	90 (6.2%)	14 (3.9%)	20 (5.5%)	25 (6.9%)	31 (8.6%)
Other	309 (21.4%)	90 (24.9%)	76 (21.0%)	65 (18.0%)	78 (21.5%)
Smoking, n (%)					
Never	716 (49.5%)	202 (56.0%)	181 (50.0%)	163 (45.0%)	170 (47.0%)
Ever	534 (36.9%)	120 (33.2%)	143 (39.5%)	141 (39.0%)	130 (35.9%)
Current	154 (10.6%)	28 (7.8%)	26 (7.2%)	47 (13.0%)	53 (14.6%)
Unknown	43 (3.0%)	11 (3.0%)	12 (3.3%)	11 (3.0%)	9 (2.5%)
Diabetes, n (%)	618 (42.7%)	153 (42.4%)	162 (44.8%)	159 (43.9%)	144 (39.8%)
Cardiovascular disease, n (%)					
Coronary artery disease	221 (15.3%)	57 (15.8%)	53 (14.6%)	59 (16.3%)	52 (14.4%)
Heart failure	98 (6.8%)	27 (7.5%)	28 (7.7%)	24 (6.6%)	19 (5.2%)
Stroke	172 (11.9%)	32 (8.9%)	43 (11.9%)	52 (14.4%)	45 (12.4%)
Peripheral artery disease	157 (10.9%)	34 (9.4%)	51 (14.1%)	33 (9.1%)	39 (10.8%)
Body mass index, kg/m^2^	23.2 (4.1)	22.5 (3.9)	23.4 (4.0)	23.2 (4.3)	23.5 (4.0)
Systolic blood pressure, mmHg	134.0 (19.2)	131.6 (20.1)	134.8 (19.4)	135.5 (18.7)	134.0 (18.2)
Diastolic blood pressure, mmHg	71.1 (12.3)	70.5 (12.9)	70.9 (11.9)	71.7 (11.7)	71.5 (12.5)
eGFR, mL/min/1.73 m^2^	17.9 (12.9, 24.7)	21.1 (14.0, 29.7)	17.4 (12.9, 23.4)	16.6 (12.1, 22.7)	17.0 (12.8, 23.2)
45	39 (2.7%)	23 (6.4%)	7 (1.9%)	3 (0.8%)	6 (1.7%)
30–44	159 (11.0%)	66 (18.3%)	26 (7.2%)	36 (9.9%)	31 (8.6%)
15–29	714 (49.3%)	171 (47.4%)	191 (52.8%)	171 (47.2%)	181 (50.0%)
15	535 (37.0%)	101 (28.0%)	138 (38.1%)	152 (42.0%)	144 (39.8%)
Hemoglobin, g/dL	9.8 (0.9)	9.7 (1.0)	9.7 (0.8)	9.8 (0.9)	9.8 (0.8)
Albumin, g/dL	3.7 (0.5)	3.7 (0.5)	3.7 (0.5)	3.8 (0.5)	3.7 (0.5)
HbA1c, %	6.1 (0.9)	6.1 (0.9)	6.1 (0.8)	6.1 (0.9)	6.1 (1.1)
High sensitive CRP, ng/dL	560.5 (215.0, 1780.0)	458.5 (171.0, 1445.0)	597.5 (254.0, 1850.0)	534.0 (208.0, 1420.0)	728.0 (290.0, 2150.0)
Urinary protein-creatinine ratio, g/gCr	1.3 (0.4, 3.1)	1.0 (0.2, 2.7)	1.3 (0.5, 3.2)	1.5 (0.4, 3.3)	1.4 (0.5, 2.8)
ARB, n (%)	853 (58.9%)	198 (54.8%)	222 (61.3%)	218 (60.2%)	215 (59.4%)
ACEi, n (%)	163 (11.3%)	46 (12.7%)	37 (10.2%)	39 (10.8%)	41 (11.3%)
iEResI	0.504 (0.252, 0.825)	0.518 (0.288, 0.840)	0.550 (0.267, 0.867)	0.495 (0.234, 0.775)	0.474 (0.242, 0.829)
Cumulative darbepoetin alpha dose, ug	130 (90, 180)	150 (90, 180)	120 (90, 180)	120 (90, 180)	150 (90, 180)
ERI-1B	4.124 (2.655, 5.882)	4.616 (2.703, 5.941)	3.756 (2.703, 5.825)	3.846 (2.655, 5.825)	4.800 (2.655, 6.061)

Data are presented as mean (SD) or median (IQR, interquartile range) unless otherwise stated

Abbreviations: ACEi, angiotensin-converting enzyme inhibitor ARBs, angiotensin II receptor blockers; CRP, C-reactive protein; eGFR, estimated glomerular filtration rate; ERI-1B, ESA hyporesponsive index-1B; HbA1c, glycated hemoglobin; iEResI, initial ESA response index; MCP-1, monocyte chemoattractant protein-1

**Fig. 1:**
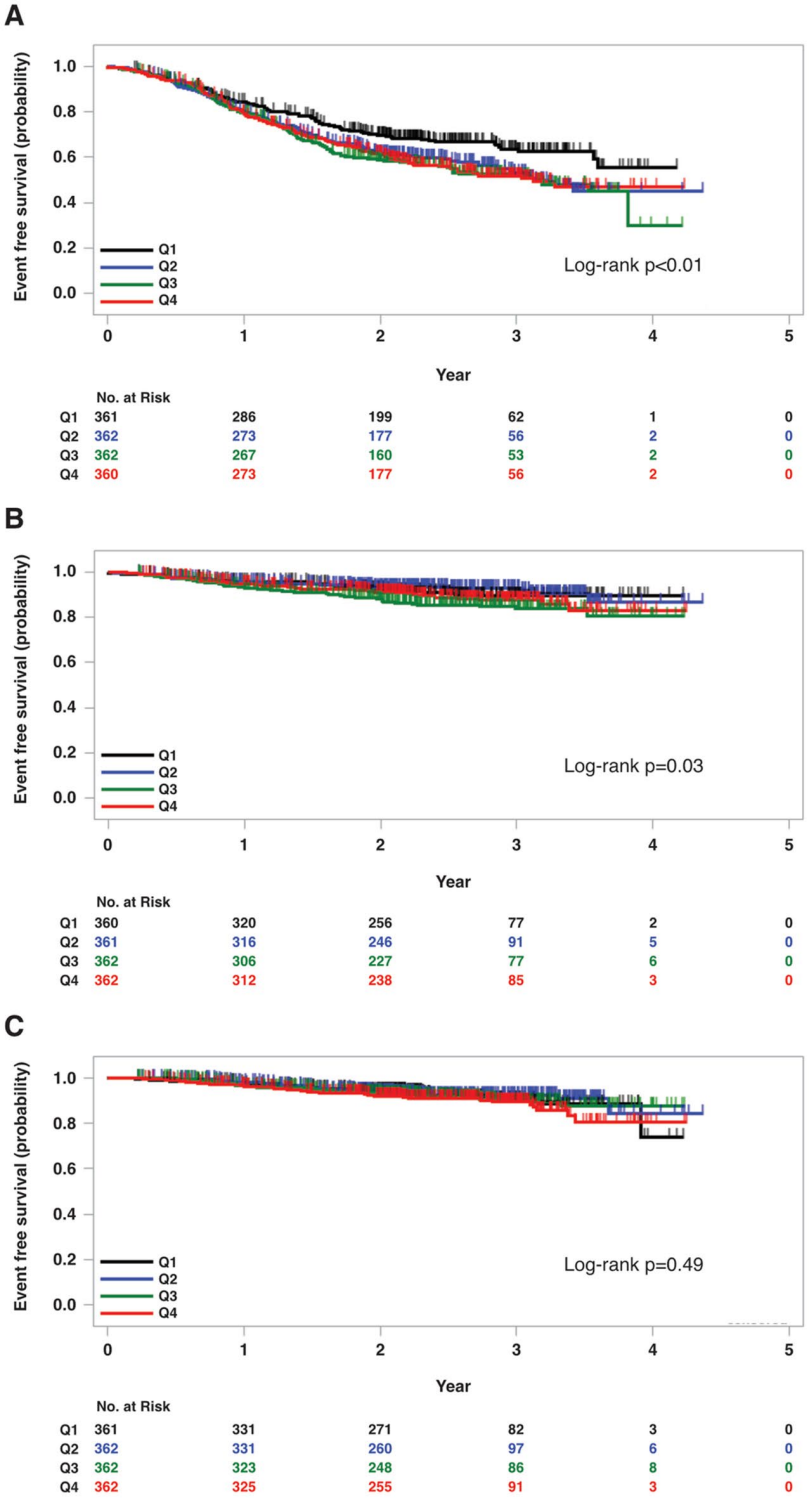
Kaplan–Meier curves for the risk of (**A**) kidney outcomes, (**B**) cardiovascular outcomes (fatal or non-fatal), and (**C**) all-cause mortality according to baseline MCP-1 levels (Q1, Q2, Q3, Q4)

### Association of MCP-1 with kidney, cardiovascular, and mortality outcomes

The Kaplan–Meier curves for each outcome are presented in Fig. 1. Participants in Q1 had the lowest risk of adverse kidney outcomes, with significant differences observed across the MCP-1 groups (log-rank *p* < 0.01). In multivariable Cox proportional hazards models, higher MCP-1 levels were associated with an increased risk of kidney outcomes after adjusting for age, sex, hemoglobin level, systolic blood pressure, body mass index, history of cardiovascular disease, and urinary protein-to-creatinine ratio (Model 2). However, this association was attenuated after additional adjustment for the baseline eGFR (Model 3) (Table 2). Breakdown of the composite kidney events (Additional File Table 1).

**Table 2:** Adjusted hazard ratios for the risk of clinical outcomes according to baseline MCP-1 levels

	Events/ *N* of total	Model 1 HR (95% CI)	*P*	Model 2 HR (95% CI)	*P*	Model 3 HR (95% CI)	*P*
**Kidney outcome**							
Q1	116/361	1.00 (reference)	-	1.00 (reference)	-	1.00 (reference)	-
Q2	150/362	1.37 (1.07, 1.74)	0.01	1.26 (0.97, 1.64)	0.08	1.00 (0.77, 1.30)	1.00
Q3	157/362	1.48 (1.17, 1.88)	< 0.01	1.42 (1.09, 1.85)	< 0.01	1.03 (0.79, 1.34)	0.83
Q4	154/360	1.42 (1.12, 1.81)	< 0.01	1.44 (1.11, 1.88)	< 0.01	1.15 (0.88, 1.50)	0.30
**Cardiovascular outcome**							
Q1	31/360	1.00 (reference)	-	1.00 (reference)	-	1.00 (reference)	-
Q2	26/361	0.84 (0.50, 1.41)	0.51	0.76 (0.43, 1.33)	0.34	0.68 (0.39, 1.20)	0.19
Q3	48/362	1.61 (1.02, 2.52)	0.04	1.55 (0.95, 2.54)	0.08	1.37 (0.83, 2.26)	0.21
Q4	38/362	1.25 (0.78, 2.00)	0.36	1.18 (0.71, 1.98)	0.53	1.04 (0.62, 1.76)	0.88
**All-cause death**							
Q1	28/361	1.00 (reference)	-	1.00 (reference)	-	1.00 (reference)	-
Q2	25/362	0.88 (0.51, 1.50)	0.63	0.83 (0.47, 1.48)	0.53	0.82 (0.46, 1.47)	0.51
Q3	29/362	1.04 (0.62, 1.75)	0.88	1.02 (0.58, 1.79)	0.94	1.01 (0.57, 1.78)	0.97
Q4	36/362	1.29 (0.79, 2.11)	0.31	1.12 (0.64, 1.93)	0.70	1.10 (0.63, 1.92)	0.74

Model 1 was unadjusted. Model 2 was adjusted for age, sex, hemoglobin, systolic blood pressure, body mass index, history of cardiovascular disease, and urinary protein-creatinine ratio. Model 3 included the same adjustments as Model 2, with the addition of baseline eGFR

Two patients with pre-existing events were excluded from the kidney outcome analysis (≥ Q3), and two patients with pre-existing events were excluded from the cardiovascular outcome analysis (< Q1 and Q1-< Q2)

Abbreviations: CI, confidence interval; HR, hazard ratio; MCP-1, monocyte chemoattractant protein-1

For cardiovascular outcomes, Kaplan–Meier analysis showed a significant difference across the MCP-1 groups (log-rank *p* = 0.03); however, this association was attenuated after adjusting for potential confounders in multivariable Cox models (Table 2). MCP-1 levels were not significantly associated with all-cause mortality in either the Kaplan–Meier analysis (log-rank, *p* = 0.49) or multivariable Cox models.

### Subgroup analyses

In the subgroup analyses (Additional File Table 2), no significant interactions were observed across the major clinical variables, including demographics, comorbidities, laboratory values, and treatment-related factors (all P for interaction > 0.05).

### eGFR decline analysis

Analysis of the annual eGFR decline using a multivariable linear mixed-effects model (Table 3) showed that participants in Q2, Q3, and Q4 experienced a significantly more rapid decline than those in Q1 (*p* < 0.05 for all comparisons). Compared to Q1, participants in Q2, Q3, and Q4 showed an additional annual eGFR decline of approximately 1.0 mL/min/1.73 m^2^. However, the interaction across the MCP-1 groups was not statistically significant (P for interaction = 0.06).

**Table 3:** Annual change in eGFR according to baseline MCP-1 levels

MCP-1 levels	Annual change in eGFR (mL/min/1.73 m^2^/year, least square mean [SE])	Difference (95% CI)	*P*	*P* for interaction
Q1	-2.61 (0.33)	(reference)	-	0.06
Q2	-3.59 (0.32)	-0.98 (-1.87, -0.09)	0.03	
Q3	-3.64 (0.33)	-1.03 (-1.93, -0.14)	0.02	
Q4	-3.62 (0.33)	-1.01 (-1.92, -0.11)	0.03	

The annual change in eGFR was estimated using a linear mixed-effects model, adjusted for baseline age, sex, hemoglobin, systolic blood pressure, eGFR, body mass index, history of cardiovascular disease, and urinary protein-creatinine ratio

Abbreviations: CI, confidence interval; eGFR, estimated glomerular filtration rate; MCP-1, monocyte chemoattractant protein-1; SE, standard error

### Predictive accuracy of MCP-1

**Fig. 2:**
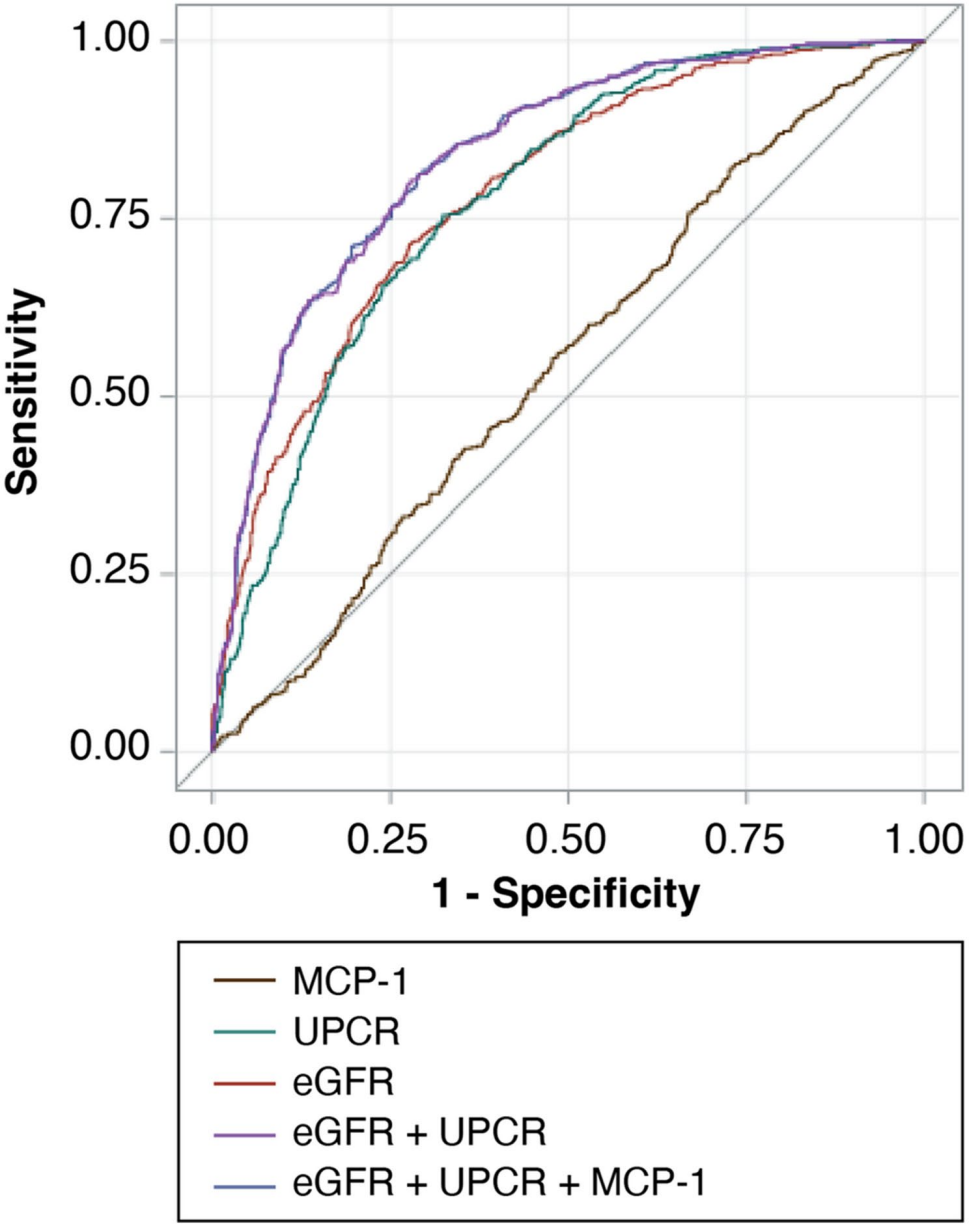
Receiver operating characteristic curves for predicting risk of kidney outcomes. The ROC curves compare the predictive accuracy of MCP-1 alone (brown line), UPCR alone (green line), eGFR alone (red line), eGFR + UPCR (purple line), and eGFR + UPCR + MCP-1 (blue line). The reference line represents random prediction. Abbreviations: eGFR, estimated glomerular filtration rate; MCP-1, monocyte chemoattractant protein-1; ROC, receiver operating characteristic; UPCR, urinary protein-creatinine ratio

The predictive accuracy of MCP-1, along with traditional risk markers such as eGFR and the urinary protein-creatinine ratio (UPCR), was further evaluated using ROC curve analysis (Fig. 2). When added to models that included eGFR and UPCR, MCP-1 levels did not improve the predictive ability for kidney outcomes (Additional File Table 3). Similar findings were observed in the discrimination statistics analysis (Additional File Table 4).

## Discussion

In this study of patients with advanced CKD (median eGFR 17.9 mL/min/1.73 m^2^), higher plasma MCP-1 levels were associated with a more rapid decline in kidney function, with an additional annual decrease of approximately 1.0 mL/min/1.73 m^2^ compared to those with the lowest MCP-1 levels. However, MCP-1 levels did not predict the progression of end-stage kidney disease, cardiovascular outcomes, or mortality. These findings suggest that MCP-1 may serve as a marker of ongoing kidney injury rather than a predictor of adverse clinical outcomes in patients with CKD, supporting its potential role in monitoring disease activity.

MCP-1 is an inflammatory chemokine that contributes to CKD progression by promoting monocyte migration and infiltration [17]. Our results support this mechanism, as higher MCP-1 levels are associated with an increased risk of decline in kidney function in patients with CKD. Specifically, participants in Q2, Q3, and Q4 experienced a more rapid eGFR decline than those in Q1. However, no clear differences were observed in the rate of eGFR decline among them. This pattern aligns with the findings of a previous large cohort study, which reported that only the highest quartile of plasma MCP-1 levels was significantly associated with adverse kidney outcomes in patients with diabetes and CKD [18]. These results suggest that MCP-1 may act as an inflammatory trigger rather than exerting a continuous, dose-dependent effect. Additionally, Q1 had a higher proportion of patients with higher baseline eGFR, which may partially explain the slower eGFR decline in this group since higher baseline eGFR is generally associated with slower decline.

An important consideration is that our study population exhibited substantially higher MCP-1 levels (median 385.9 pg/mL) compared to other CKD cohorts, likely reflecting our more advanced CKD stage (median eGFR 17.9 mL/min/1.73 m²) and the progressive increase in MCP-1 with CKD severity [12, 18]. Using study-specific quartiles in a uniformly high-MCP-1 population may have limited our ability to detect outcome associations, as even our lowest quartile would be considered elevated in cohorts with less advanced CKD. While prior research in early-stage CKD demonstrated that serum MCP-1 was associated with albuminuria and eGFR in patients with preserved kidney [19], the prognostic utility of MCP-1 may be attenuated in populations with advanced CKD and high baseline MCP-1 levels.

The differing relationships between MCP-1 levels and the decline in kidney function warrant careful interpretation. Although elevated MCP-1 levels were associated with a more rapid eGFR decline, they did not predict composite kidney outcomes indicative of advanced kidney failure. This discrepancy may be explained by the role of MCP-1 in the pathogenesis of CKD. MCP-1 is involved in early kidney inflammation and fibrosis [10], contributing to the progressive decline in eGFR. It is important to note that prior studies demonstrating strong associations between MCP-1 and kidney disease activity primarily measured urinary MCP-1, which reflects local tubulointerstitial inflammation and correlates with intrarenal inflammatory cell infiltration [9, 10], whereas our measurement of plasma MCP-1 captures systemic inflammatory status. In later CKD stages, other factors, such as hemodynamic changes, may play a more dominant role, potentially diminishing the impact of MCP-1 [20].

In the univariable analysis, higher MCP-1 levels were associated with cardiovascular outcomes; however, these relationships lost significance after adjusting for traditional cardiovascular risk factors. This finding aligns with previous studies reporting similar results in patients with CKD [13]. In CKD, the pathogenesis of cardiovascular disease involves multiple factors beyond inflammation, including medial arterial calcification, which develops differently from the intimal atherosclerotic calcified plaques observed in the general population [13]. Additionally, uremic toxins, mineral and bone disorders, and other CKD-specific factors likely influence cardiovascular outcomes [21]. Therefore, MCP-1 provides limited additional predictive value for cardiovascular outcomes in CKD beyond traditional risk factors. We also did not observe significant associations between MCP-1 and all-cause mortality. This discrepancy with some previous reports [13] may reflect our population’s more advanced CKD with uniformly elevated MCP-1 levels, which may have limited discriminative ability for mortality risk, combined with our shorter follow-up period.

### Strengths and limitations

This study had several strengths. First, its prospective design enabled an accurate evaluation of the relationship between baseline MCP-1 levels and subsequent kidney function decline through repeated eGFR measurements over time. Second, the inclusion of 1,447 participants from multiple centers in Japan allowed for the analysis of a relatively large cohort of patients with CKD. Third, by systematically capturing both the trajectory of kidney function and major clinical events within the same cohort, we examined the relationship between MCP-1 levels and different aspects of CKD progression.

This study had some limitations. First, the study population was restricted to patients with renal anemia who initiated treatment. A substantial proportion of patients had advanced CKD (eGFR < 15 mL/min/1.73 m²), which may limit the applicability of our findings to the broader CKD population. Second, our analysis relied solely on baseline MCP-1 measurements and did not assess the impact of longitudinal changes in MCP-1 levels on renal outcomes. Third, the relatively short follow-up period may have limited the study’s ability to detect associations with clinical outcomes, particularly mortality and hard renal outcomes, such as end-stage renal disease. Fourth, as this study was conducted at a Japanese institution, the generalizability of our findings to populations with different ethnic backgrounds requires further investigation.

## Conclusion

In summary, higher plasma MCP-1 levels are associated with a more rapid decline in kidney function but do not predict the composite outcome of kidney failure in patients with advanced CKD. MCP-1 was not associated with cardiovascular outcomes or mortality in this population. These findings suggest that MCP-1 is a marker of ongoing kidney injury rather than a predictor of adverse outcomes. Further studies are needed to determine whether interventions targeting the MCP-1 pathway improve kidney function in patients with CKD.

## Supplementary Information

Below is the link to the electronic supplementary material.

Supplementary Material 1

## Data Availability

The datasets used and/or analysed during the current study are available from the corresponding author upon reasonable request.

## Abbreviations

ACEi: Angiotensin-converting enzyme inhibitor
ARB: Angiotensin II receptor blocker
BMI: Body mass index
CCL2: C-C motif chemokine ligand 2
CI: Confidence interval
CKD: Chronic kidney disease
CRP: C-reactive protein
CVD: Cardiovascular disease
eGFR: Estimated glomerular filtration rate
ERI-1B: ESA hyporesponsive index-1B
ESA: Erythropoiesis-stimulating agent
ESRD: End-stage renal disease
HbA1c: Glycated hemoglobin
HR: Hazard ratio
IDI: Integrated discrimination improvement
iEResI: Initial ESA Response Index
IQR: Interquartile range
MCP-1: Monocyte chemoattractant protein-1
NRI: Net Reclassification improvement
ROC: Receiver operating characteristic
SD: Standard deviation
SE: Standard error
UPCR: Urinary protein-creatinine ratio
