# Supplementary material for: Plasma monocyte chemoattractant protein-1 and the risk of kidney and cardiovascular outcomes in people with chronic kidney disease: results from the BRIGHTEN study
Source: BMC Nephrol. 2025 Dec 29;26:705. doi: 10.1186/s12882-025-04633-y (PMC12751990; doi:10.1186/s12882-025-04633-y)
Supplement: Supplementary file 1 — Supplementary Material 1 [file 12882_2025_4633_MOESM1_ESM.docx]

**Additional File**

**Additional File Table 1.** Adjusted hazard ratios for the risk of clinical outcomes according to baseline MCP-1 levels (components of primary outcomes)

|  | **Events/ N of total** | **Model 1**  **HR (95% CI)** | **P** | **Model 2**  **HR (95% CI)** | **P** | **Model 3**  **HR (95% CI)** | **P** |
| --- | --- | --- | --- | --- | --- | --- | --- |
| **Dialysis** | |  |  |  |  |  |  |
| Q1 | 85/361 | 1.00  (reference) | - | 1.00  (reference) | - | 1.00  (reference) | - |
| Q2 | 125/362 | 1.57  (1.19, 2.07) | <0.01 | 1.43  (1.06, 1.93) | 0.02 | 1.19  (0.88, 1.61) | 0.25 |
| Q3 | 134/362 | 1.72  (1.31, 2.26) | <0.01 | 1.69  (1.25, 2.27) | <0.01 | 1.23  (0.91, 1.66) | 0.18 |
| Q4 | 131/360 | 1.66  (1.26, 2.18) | <0.01 | 1.69  (1.25, 2.28) | <0.01 | 1.41  (1.04, 1.91) | 0.03 |
|  |  |  |  |  |  |  |  |
| **Transplant** | |  |  |  |  |  |  |
| Q1 | 8/361 | 1.00  (reference) | - | 1.00  (reference) | - | 1.00  (reference) | - |
| Q2 | 4/362 | 0.50  (0.15, 1.67) | 0.26 | 0.77  (0.22, 2.75) | 0.69 | 0.72  (0.20, 2.59) | 0.62 |
| Q3 | 5/362 | 0.64  (0.21, 1.96) | 0.44 | 1.12  (0.35, 3.66) | 0.85 | 0.96  (0.29, 3.13) | 0.94 |
| Q4 | 2/360 | 0.25  (0.05, 1.18) | 0.08 | 0.45  (0.09, 2.27) | 0.36 | 0.37  (0.07, 1.84) | 0.22 |
|  |  |  |  |  |  |  |  |
| **50% eGFR decline** | |  |  |  |  |  |  |
| Q1 | 47/361 | 1.00  (reference) | - | 1.00  (reference) | - | 1.00  (reference) | - |
| Q2 | 58/362 | 1.26  (0.86, 1.85) | 0.25 | 1.16  (0.76, 1.75) | 0.49 | 1.08  (0.71, 1.63) | 0.74 |
| Q3 | 56/362 | 1.25  (0.85, 1.85) | 0.25 | 1.10  (0.72, 1.67) | 0.67 | 0.99  (0.65, 1.52) | 0.98 |
| Q4 | 55/360 | 1.20  (0.81, 1.77) | 0.36 | 1.09  (0.71, 1.67) | 0.71 | 1.00  (0.65, 1.54) | 1.00 |
|  |  |  |  |  |  |  |  |
| **eGFR less or equal to 6** **mL/min/1.73m^2^** | |  |  |  |  |  |  |
| Q1 | 37/361 | 1.00  (reference) | - | 1.00  (reference) | - | 1.00  (reference) | - |
| Q2 | 52/362 | 1.43  (0.94, 2.18) | 0.10 | 1.26  (0.81, 1.99) | 0.31 | 1.10  (0.70, 1.74) | 0.68 |
| Q3 | 51/362 | 1.44  (0.94, 2.19) | 0.09 | 1.50  (0.96, 2.35) | 0.07 | 1.15  (0.73, 1.81) | 0.54 |
| Q4 | 50/360 | 1.40  (0.92, 2.14) | 0.12 | 1.48  (0.94, 2.32) | 0.09 | 1.29  (0.81, 2.04) | 0.29 |

**Additional File Table 2.** Relationships between baseline MCP-1 and risk of kidney outcomes stratified by participant characteristics

| **Subgroup** | **Baseline**  **MCP-1** | **N of events/total** | **Multivariable HR**  **(95% CI)** | **P** | **P for interaction** |
| --- | --- | --- | --- | --- | --- |
| **Age, years** |  |  |  |  | 0.97 |
| <60 | Q1 | 29/66 | 1.00 (reference) | - |  |
|  | Q2 | 29/54 | 1.20 (0.72, 2.02) | 0.48 |  |
|  | Q3 | 34/54 | 1.35 (0.82, 2.22) | 0.25 |  |
|  | Q4 | 24/42 | 1.24 (0.72, 2.14) | 0.43 |  |
| ≥60 | Q1 | 71/237 | 1.00 (reference) | - |  |
|  | Q2 | 105/262 | 1.30 (0.96, 1.77) | 0.09 |  |
|  | Q3 | 104/254 | 1.43 (1.05, 1.93) | 0.02 |  |
|  | Q4 | 107/254 | 1.46 (1.08, 1.98) | 0.02 |  |
| **Sex** |  |  |  |  | 0.57 |
| Men | Q1 | 65/162 | 1.00 (reference) | - |  |
|  | Q2 | 89/188 | 1.24 (0.90, 1.71) | 0.19 |  |
|  | Q3 | 99/185 | 1.50 (1.10, 2.06) | 0.01 |  |
|  | Q4 | 100/180 | 1.57 (1.15, 2.15) | 0.01 |  |
| Women | Q1 | 35/141 | 1.00 (reference) | - |  |
|  | Q2 | 45/128 | 1.32 (0.84, 2.06) | 0.23 |  |
|  | Q3 | 39/123 | 1.27 (0.80, 2.01) | 0.32 |  |
|  | Q4 | 31/116 | 1.17 (0.72, 1.90) | 0.53 |  |
| **Smoking** |  |  |  |  | 0.06 |
| Never | Q1 | 54/173 | 1.00 (reference) | - |  |
|  | Q2 | 52/158 | 0.91 (0.61, 1.34) | 0.62 |  |
|  | Q3 | 53/138 | 1.20 (0.81, 1.76) | 0.36 |  |
|  | Q4 | 50/141 | 1.24 (0.84, 1.83) | 0.28 |  |
| Ever | Q1 | 36/99 | 1.00 (reference) | - |  |
|  | Q2 | 66/131 | 1.54 (1.03, 2.32) | 0.04 |  |
|  | Q3 | 58/125 | 1.49 (0.98, 2.26) | 0.06 |  |
|  | Q4 | 56/104 | 1.95 (1.27, 2.97) | <0.01 |  |
| Current | Q1 | 8/23 | 1.00 (reference) | - |  |
|  | Q2 | 14/19 | 2.74 (1.15, 6.56) | 0.02 |  |
|  | Q3 | 24/36 | 2.43 (1.08, 5.47) | 0.03 |  |
|  | Q4 | 23/47 | 1.46 (0.65, 3.30) | 0.36 |  |
| **Diabetes** |  |  |  |  | 0.45 |
| Yes | Q1 | 46/129 | 1.00 (reference) | - |  |
|  | Q2 | 65/143 | 1.07 (0.73, 1.58) | 0.72 |  |
|  | Q3 | 64/137 | 1.22 (0.83, 1.79) | 0.32 |  |
|  | Q4 | 55/117 | 1.14 (0.76, 1.69) | 0.53 |  |
| No | Q1 | 54/174 | 1.00 (reference) | - |  |
|  | Q2 | 69/173 | 1.46 (1.02, 2.09) | 0.04 |  |
|  | Q3 | 74/171 | 1.63 (1.15, 2.32) | 0.01 |  |
|  | Q4 | 76/179 | 1.75 (1.23, 2.49) | <0.01 |  |
| **Etiology of CKD** |  |  |  |  | 0.87 |
| Diabetic nephropathy | Q1 | 34/82 | 1.00 (reference) | - |  |
|  | Q2 | 45/92 | 1.03 (0.66, 1.61) | 0.91 |  |
|  | Q3 | 48/88 | 1.27 (0.82, 1.98) | 0.29 |  |
|  | Q4 | 39/78 | 1.02 (0.64, 1.63) | 0.92 |  |
| Chronic glomerulonephritis | Q1 | 23/68 | 1.00 (reference) | - |  |
|  | Q2 | 37/83 | 1.26 (0.75, 2.12) | 0.39 |  |
|  | Q3 | 32/69 | 1.38 (0.80, 2.36) | 0.25 |  |
|  | Q4 | 32/65 | 1.63 (0.95, 2.79) | 0.08 |  |
| Nephrosclerosis | Q1 | 18/71 | 1.00 (reference) | - |  |
|  | Q2 | 24/62 | 1.69 (0.91, 3.12) | 0.09 |  |
|  | Q3 | 28/80 | 1.62 (0.89, 2.93) | 0.11 |  |
|  | Q4 | 27/75 | 1.74 (0.96, 3.17) | 0.07 |  |
| Polycystic kidney disease | Q1 | 3/12 | 1.00 (reference) | - |  |
|  | Q2 | 5/15 | 2.31 (0.55, 9.72) | 0.25 |  |
|  | Q3 | 8/20 | 2.61 (0.69, 9.92) | 0.16 |  |
|  | Q4 | 10/19 | 4.32 (1.18, 15.80) | 0.03 |  |
| Others | Q1 | 22/70 | 1.00 (reference) | - |  |
|  | Q2 | 23/64 | 1.17 (0.65, 2.11) | 0.60 |  |
|  | Q3 | 22/51 | 1.39 (0.77, 2.51) | 0.28 |  |
|  | Q4 | 23/59 | 1.43 (0.79, 2.57) | 0.24 |  |

(Continued)

| Subgroup | Baseline  MCP-1 | N of events/total | Multivariable HR  (95% CI) | P | P for interaction |
| --- | --- | --- | --- | --- | --- |
| **History of CVD** |  |  |  |  | 0.46 |
| Yes | Q1 | 20/86 | 1.00 (reference) | - |  |
|  | Q2 | 41/101 | 1.77 (1.04, 3.03) | 0.04 |  |
|  | Q3 | 35/84 | 1.80 (1.04, 3.14) | 0.04 |  |
|  | Q4 | 40/94 | 2.03 (1.19, 3.48) | 0.01 |  |
| No | Q1 | 80/217 | 1.00 (reference) | - |  |
|  | Q2 | 93/215 | 1.13 (0.83, 1.53) | 0.43 |  |
|  | Q3 | 103/224 | 1.32 (0.98, 1.78) | 0.06 |  |
|  | Q4 | 91/202 | 1.29 (0.95, 1.75) | 0.10 |  |
| **eGFR, mL/min/1.73 m^2^** |  |  |  |  | 0.52 |
| <15 | Q1 | 54/84 | 1.00 (reference) | - |  |
|  | Q2 | 82/123 | 1.01 (0.72, 1.44) | 0.94 |  |
|  | Q3 | 84/133 | 1.05 (0.74, 1.48) | 0.80 |  |
|  | Q4 | 87/120 | 1.23 (0.87, 1.73) | 0.25 |  |
| ≥15 | Q1 | 46/219 | 1.00 (reference) | - |  |
|  | Q2 | 52/193 | 1.23 (0.83, 1.84) | 0.30 |  |
|  | Q3 | 54/175 | 1.43 (0.96, 2.12) | 0.08 |  |
|  | Q4 | 44/176 | 1.20 (0.79, 1.82) | 0.39 |  |
| **UPCR, g/gCr** |  |  |  |  | 0.24 |
| <1 | Q1 | 18/148 | 1.00 (reference) | - |  |
|  | Q2 | 28/132 | 1.94 (1.07, 3.52) | 0.03 |  |
|  | Q3 | 27/122 | 1.96 (1.08, 3.56) | 0.03 |  |
|  | Q4 | 27/118 | 2.36 (1.29, 4.29) | <0.01 |  |
| ≥1 | Q1 | 82/155 | 1.00 (reference) | - |  |
|  | Q2 | 106/184 | 1.09 (0.81, 1.45) | 0.58 |  |
|  | Q3 | 111/186 | 1.33 (1.00, 1.77) | 0.05 |  |
|  | Q4 | 104/178 | 1.23 (0.92, 1.65) | 0.16 |  |
| **ACEi or ARB use** |  |  |  |  | 0.90 |
| Yes | Q1 | 66/193 | 1.00 (reference) | - |  |
|  | Q2 | 100/229 | 1.20 (0.88, 1.65) | 0.25 |  |
|  | Q3 | 94/208 | 1.38 (1.01, 1.90) | 0.05 |  |
|  | Q4 | 95/208 | 1.37 (1.00, 1.89) | 0.05 |  |
| No | Q1 | 34/110 | 1.00 (reference) | - |  |
|  | Q2 | 34/87 | 1.46 (0.90, 2.36) | 0.13 |  |
|  | Q3 | 44/100 | 1.50 (0.96, 2.36) | 0.08 |  |
|  | Q4 | 36/88 | 1.65 (1.03, 2.66) | 0.04 |  |
| **ERI-1B** |  |  |  |  | 0.87 |
| <5.3 | Q1 | 43/166 | 1.00 (reference) | - |  |
|  | Q2 | 69/179 | 1.42 (0.96, 2.08) | 0.08 |  |
|  | Q3 | 69/170 | 1.51 (1.02, 2.22) | 0.04 |  |
|  | Q4 | 66/159 | 1.64 (1.11, 2.41) | 0.01 |  |
| ≥5.3 | Q1 | 47/106 | 1.00 (reference) | - |  |
|  | Q2 | 53/100 | 1.26 (0.84, 1.87) | 0.27 |  |
|  | Q3 | 53/109 | 1.28 (0.86, 1.90) | 0.22 |  |
|  | Q4 | 53/104 | 1.30 (0.87, 1.93) | 0.20 |  |
| **Cumulative dalbepoetin alpha dose, μg** |  |  |  |  | 0.58 |
| <120 | Q1 | 26/108 | 1.00 (reference) | - |  |
|  | Q2 | 49/127 | 1.51 (0.94, 2.44) | 0.09 |  |
|  | Q3 | 45/105 | 1.75 (1.08, 2.84) | 0.02 |  |
|  | Q4 | 33/97 | 1.44 (0.86, 2.42) | 0.17 |  |
| ≥120 | Q1 | 74/195 | 1.00 (reference) | - |  |
|  | Q2 | 85/189 | 1.18 (0.86, 1.61) | 0.32 |  |
|  | Q3 | 93/203 | 1.28 (0.94, 1.74) | 0.13 |  |
|  | Q4 | 98/199 | 1.42 (1.04, 1.92) | 0.03 |  |

Models were adjusted for age, sex, hemoglobin, systolic blood pressure, body mass index, history of cardiovascular disease, and protein-creatinine ratio.

Abbreviations: ACEi, angiotensin-converting enzyme inhibitor ARBs, angiotensin II receptor blocker; CVD, cardiovascular disease; eGFR, estimated glomerular filtration rate; ERI-1B, ESA hyporesponsive index-1B; MCP-1, monocyte chemoattractant protein-1; UPCR, Urinary protein-creatinine ratio.

**Additional File Table 3.** Model comparison for predicting risk of kidney outcomes.

| **Model** | **Optimum**  **cut-off value** | **Youden’s**  **index** | **C-statistic**  **(95% CI)** | **Difference**  **(95% CI)** | **P** |
| --- | --- | --- | --- | --- | --- |
| MCP-1 | 321.4  (pg/mL) | 0.092 | 0.54  (0.51, 0.58) | -0.29  (-0.33, -0.26) | <0.01 |
| UPCR | 1.32  (g/gCr) | 0.431 | 0.78  (0.75, 0.80) | -0.06  (-0.08, -0.04) | <0.01 |
| eGFR | 16.9  (mL/min/1.73 m^2^) | 0.437 | 0.79  (0.76, 0.81) | -0.05  (-0.07, -0.04) | <0.01 |
| eGFR  + UPCR | - | 0.523 | 0.84  (0.82, 0.86) | 0.00  (0.00, 0.00) | 0.54 |
| eGFR  + UPCR  + MCP-1 | - | 0.522 | 0.84  (0.82, 0.86) | (Reference) | (Reference) |

Abbreviations: eGFR, estimated glomerular filtration rate; MCP-1, monocyte chemoattractant protein-1; UPCR, Urinary protein-creatinine ratio.

**Additional File Table 4.** Discrimination statistics for predicting risk of kidney outcomes.

| **Model** | **NRI**  **(95% CI)** | **P** | **IDI**  **(95% CI)** | **P** |
| --- | --- | --- | --- | --- |
| MCP-1 | -1.03  (-1.12, -0.94) | <0.01 | -0.32  (-0.35, -0.30) | <0.01 |
| UPCR | -0.79  (-0.89, -0.69) | <0.01 | -0.17  (-0.19, -0.15) | <0.01 |
| eGFR | -0.71  (-0.81, -0.61) | <0.01 | -0.09  (-0.11, -0.08) | <0.01 |
| eGFR+UPCR | -0.10  (-0.20, 0.01) | 0.08 | 0.00  (0.00, 0.00) | 0.30 |
| eGFR + UPCR + MCP-1 | (Reference) | (Reference) | (Reference) | (Reference) |

Abbreviations: eGFR, estimated glomerular filtration rate; IDI, integrated discrimination improvement; MCP-1, monocyte chemoattractant protein-1; NRI, net reclassification improvement; UPCR, Urinary protein-creatinine ratio.

**
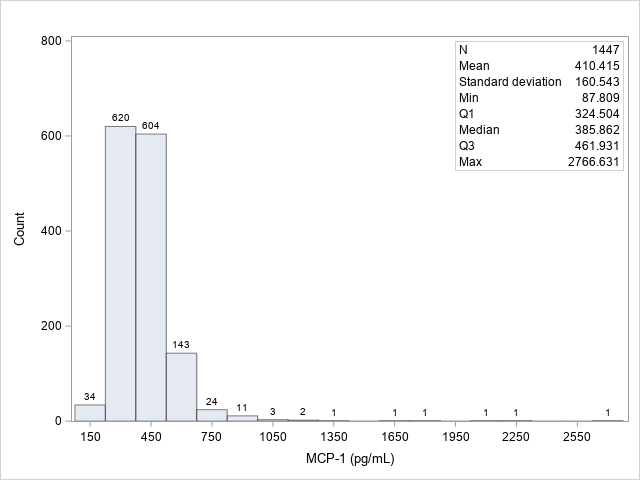
**

**Additional File Figure 1.** Distribution of baseline MCP-1 levels


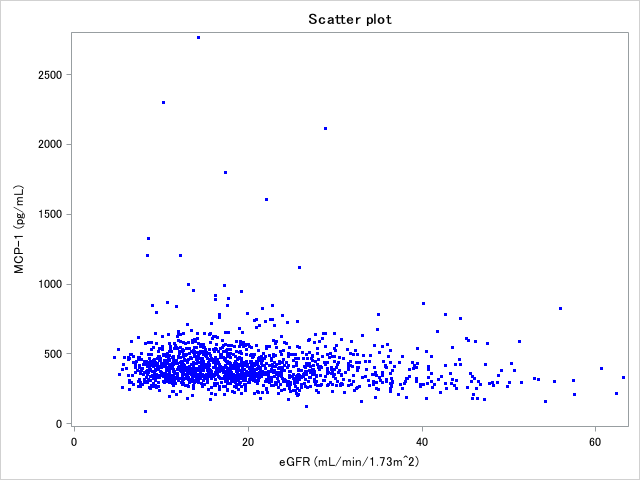


**Additional File Figure 2.** Correlation between baseline eGFR and MCP-1

Correlation between baseline eGFR and MCP-1 levels. Spearman's correlation coefficient (r = -0.14, p < 0.001). Each dot represents an individual participant (n = 1,447).
